# Impact of Oncology Drug Review Times on Public Funding Recommendations

**DOI:** 10.3390/curroncol30080558

**Published:** 2023-08-18

**Authors:** Marya Hussain, Chelsea Wong, Eddy Taguedong, Saurav Verma, Md Mahsin, Safiya Karim, Richard Lee-Ying, Doreen A. Ezeife

**Affiliations:** 1 Department of Oncology, University of Calgary, Calgary, AB T2N 4N2, Canada; safiya.karim@ahs.ca (S.K.); richard.lee-ying@ahs.ca (R.L.-Y.); doreen.ezeife@ahs.ca (D.A.E.); 2 Faculty of Arts, Department of Psychology, University of Calgary, Calgary, AB T2N 1N4, Canada; chelsea.wong@ucalgary.ca; 3 Department of Medicine, McGill University, Montreal, QC H3A 0G4, Canada; edouarda.taguedong@mail.mcgill.ca; 4 London Regional Cancer Program, Department of Medical Oncology, London Health Sciences Centre, London, ON N6A 5W9, Canada; saurav.verma@lhsc.on.ca; 5 Precision Oncology Hub, Alberta Health Services, Calgary, AB T2N 4Z6, Canada; md.mahsin@ahs.ca

**Keywords:** oncology drug review, oncology drugs, Canadian Agency for Drugs and Technologies in Health (CADTH), drug approval time

## Abstract

**Simple Summary:**

Cancer is one of the leading causes of death in Canada and worldwide, and there is ongoing discovery of new cancer medicines. However, before these drugs are publicly funded, they undergo a rigorous review process. In a single-payer health system, additional reviews are necessary to balance clinical benefit with cost effectiveness, which can cause delays in the public availability of these lifesaving treatments. Our study aimed to examine the factors that impact these drug review times and, ultimately, public funding recommendations. Interestingly, we found that longer drug review times (time from Health Canada approval to pCODR final recommendations) were associated with negative funding recommendations, with drugs for lung and gastrointestinal cancers undergoing more lengthy review processes.

**Abstract:**

New oncology drugs undergo detailed review prior to public funding in a single-payer healthcare system. The aim of this study was to assess how cancer drug review times impact funding recommendations. Drugs reviewed by the pan-Canadian Oncology Drug Review (pCODR) between the years 2012 and 2020 were included. Data were collected including Health Canada approval dates, initial and final funding recommendations, treatment intent, drug class, clinical indications, and incremental cost-effectiveness ratios (ICER). Univariable and multivariable analyses were used to determine the association between funding recommendations and review times. Of the 164 applications submitted, 130 received a positive final recommendation. Median time from Health Canada (HC) approval to final recommendation was longer for drugs indicated for the treatment of gastrointestinal (GI) and lung cancer compared to breast, genitourinary (GU), and other tumours (205 vs. 198 vs. 111 vs. 129 vs. 181 days, respectively; Kruskal–Wallis *p* = 0.0312). Drugs with longer review times were more likely to receive a negative pCODR recommendation, even when adjusting for tumour type, drug class, and intent of therapy (157 vs. 298 days; Wilcoxon *p* = 0.0003, OR 1.002 95% CI [1.000–1.004].). There was no association between funding recommendation and tumour type or class of drug. The exploration of factors associated with variance in review times will be important in ensuring timely patient access to cancer drugs.

## 1. Introduction

Cancer is one of the leading causes of death worldwide. As the oncology treatment landscape rapidly evolves with the emergence of novel drugs, timely access to these cancer treatments can improve cancer mortality. In countries with a public healthcare system such as Canada, the complexity of drug approval pathways and lengthy drug review processes can lead to delays in public access to novel medications. As the cost of cancer drugs rises, careful consideration is increasingly needed when selecting which drugs should be publicly reimbursed. Prior data have shown that delays in public funding can result in up to 40,000 potential life-years lost, largely driven by colorectal and lung cancer patients awaiting public access to cancer medicines [1].

Drug review timelines in public healthcare systems like Canada are longer than in private healthcare systems like the United States [2]. For a new cancer drug to make it from a successful clinical trial to a publicly reimbursed standard of care, there are multiple regulatory, evaluation, and funding steps [3]. After submission to and approval of the drug by Health Canada (HC), the cancer medicine is reviewed by the Canadian Agency for Drugs and Technologies in Health (CADTH). CADTH, through the pan-Canadian Oncology Drug Review (pCODR) process, evaluates clinical effectiveness, cost-effectiveness information, and patient perspectives on new cancer drugs and uses this evaluation to provide cancer drug funding recommendations to Canadian provinces and territories [4,5]. When reviewing new drug applications, HC sets a target of 205 days for priority reviews and 345 days for standard reviews, while CADTH-pCODR sets a target of 180 days for health technology assessment (HTA) reviews [6].

Cancer public funding decisions necessitate a prudent decision-making process that can be lengthy. During this time, cancer patients may not have public access to life-saving cancer treatments. The purpose of this study was to examine factors that impact pCODR funding recommendations and drug review times.

## 2. Materials and Methods

Cancer drugs that were reviewed by Canada’s health technology assessment body, pCODR, with a final recommendation date from April 2012 (i.e., the first drugs reviewed by pCODR) to November 2020 were included in this study (*n* = 164). All drugs reviewed by pCODR were studied, including drugs for lung, breast, gastrointestinal, genitourinary, hematologic, and gynecological cancers. 

The following information was obtained from the CADTH website: Health Canada approval date; drug class (cytotoxic chemotherapy, targeted therapy, immunotherapy, or others based on mechanism of action); treatment intent (neoadjuvant/adjuvant/curative or palliative); clinical indication (based on tumour type); initial and final recommendation (not recommended, conditionally recommended, recommended); initial and final recommendation date; notice of compliance (NOS) date; pCODR submission date; length of review; and incremental cost-effectiveness ratios (ICER).

For each drug, time intervals were calculated for the time from initial Health Canada approval to final recommendations by pCODR. The median time was also calculated for the time from pCODR submission to final recommendations. In Canada, breast, prostate, and colorectal cancers account for nearly half of all 25-year prevalent cancers (25-year prevalence refers to cases diagnosed between 1993 and 2017) [7], whereas lung cancer has the highest mortality. We hypothesized that those cancers with higher prevalence or mortality may potentially have an impact on review times. Hence, we analyzed the association of length of review by cancer type. We also assessed the association between the duration of the review and final recommendations and the association between drug class and final recommendations. 

Statistical analyses compared mean and median values for the above-defined time intervals. Comparisons were made using Wilcoxon rank-sum tests and Kruskal–Wallis analyses of variance for nonparametric data. All tests of significance (*p* values) were 2-sided. Statistical significance was represented by *p* < 0.05. Comparisons were also made using quantile regression analysis. Multiple linear regression models were used to determine the association between funding recommendations and review timelines for different types of cancer while adjusting for drug class, intent of therapy, and final drug recommendation categories. 

## 3. Results

A total of 164 cancer drugs underwent pCODR review between April 2012 and November 2020. Of these, 24 (15%) were for lung cancer, 16 (10%) for breast cancer, 18 (11%) for GI cancers, 17 (10%) for GU cancers, and 89 (60%) for other cancers, which included skin, hematologic, and gynecological cancers. Of the 164 drugs that were reviewed, 117 (71%) received conditional approval, 13 (8%) received full recommendation, and 34 (21%) were not recommended.

There were 154 drugs with Health Canada approval dates available. The median time for all 154 drugs was 176 days. Median time from Health Canada (HC) approval to final recommendation was longer for drugs indicated for the treatment of gastrointestinal (GI) and lung cancers compared to breast, genitourinary (GU), and other tumours (205 vs. 198 vs. 111 vs. 129 vs. 181 days, respectively; Kruskal–Wallis *p* = 0.0312 (Table 1)). On quantile regression, it was noted that the mean time from HC approval to final recommendation was 1.74 times longer for lung cancer compared to breast cancer; *p* = 0.03, IQR 171 (Supplementary Table S1). 

The median time from pCODR submission to final recommendation for all 164 drugs was 207 days. There was a tendency towards longer pCODR review times for lung and breast cancer drugs compared to other cancer types, although these results were not statistically significant (Table 2). On quantile regression, a trend was again noted towards longer mean review times for lung cancer (14.4 days compared to breast cancer; *p* = 0.34) and longer review times for breast cancer compared to all other cancer groups. These findings were, however, not statistically significant (Supplementary Table S2).

**Figure 1:**
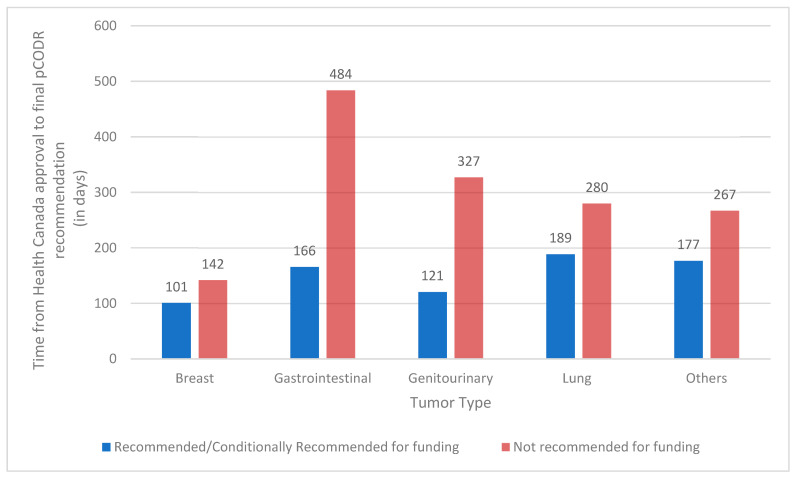
Median time from Health Canada approval to final recommendation (in days) by tumour type and funding recommendations. pCODR, pan-Canadian Oncology Drug Review.

We observed that the median review time (time from Health Canada approval to final recommendation) was nearly double for drugs that eventually received a negative pCODR recommendation as compared to those with a positive recommendation (median 298 days vs. 157 days; Wilcoxon *p* = 0.0003.). This remained true on multivariable analysis when adjusting for tumour type, drug class, and intent of therapy (OR 1.002 95% CI [1.000–1.004]). On quantile regression, drugs that were recommended needed 45% less time than those that were not recommended (*p* < 0.001) (Supplementary Table S1). Figure 1 shows the length of review (median time from Health Canada approval to final recommendation in days) by tumour type and funding recommendations. Across all tumour sites, a longer length of review was associated with an eventual negative final recommendation.

An analysis of oncology drugs by their mechanism of action (i.e., cytotoxic chemotherapy; targeted therapy; immunotherapy; and other mechanisms of action) revealed no association between the class of drug and the time from Health Canada to pCODR final recommendation (Kruskal–Wallis *p* value = 0.24) or time from pCODR submission to final recommendation (Kruskal–Wallis *p* value = 0.78). Additionally, the class of drug did not have an impact on pCODR’s final recommendation, either positively or negatively (Fisher’s Exact *p* value = 0.55) (Table 3).

## 4. Discussion

In the last decade, there has been a swift increase in novel oncology drugs alongside increasing drug costs [8,9]. The annual growth of cancer medicines has been over 20% in recent years [10]. In this accelerated growth climate, regulatory authorities must ensure the sustainability of single-payer healthcare systems through careful consideration of cancer drugs undergoing review for public funding. We found that cancer drugs with longer review times were more likely to receive a negative recommendation for funding. The reason for this finding may be that the drugs that do not get approved have insufficient data or missing data that are important for pCODR deliberation (for example, QoL data), and when requests for more data are made to applying companies, they are either delayed, not provided, or insufficient to address the pCODR concern, leading to those drugs ultimately being rejected. Moreover, some sponsors’ nonparticipation in the Health Canada information-sharing process could result in CADTH not having advance notice of changes that could impact their decisions; for instance, changes to the indicated patient population [11]. A study evaluating the relative importance of different pCODR criteria on the likelihood of a drug receiving a positive, negative, or conditional recommendation suggested that pCODR’s decision to reject versus approve a submission was driven almost exclusively by the clinical profile (quality of the clinical evidence, relative survival gains and the incidence of adverse events) and the consideration of alternatives [12].

In our analyses, medicines treating GI and lung cancer were more likely to undergo lengthy review processes compared to breast, GU, and other cancers. Interestingly, the median time from Health Canada approval to pCODR final recommendation was significantly longer for GI and lung drugs, but there was no difference in the review time from pCODR submission to final recommendation for the different tumour groups. This highlights a possible area of investigation to assess for factors that lead to a delay in submission to pCODR after a medication has received a Health Canada notice of compliance (NOC). Since March 2018, to address the issue of health technology assessment delays, a change was made so that all pCODR submissions could be submitted up to 6 months ahead of the anticipated notice of compliance date [13,14]. Parallel processes like pre-NOC submissions represent an opportunity both in terms of time gained and the probability of listing [15].

Although health technology assessments can be delayed for several administrative reasons, the impact of variables such as drug cost-effectiveness and the magnitude of clinical benefit is unknown. Our study had insufficient data to study the impact of economic considerations on drug review timelines. It is plausible that drugs with a small magnitude of clinical benefit undergo lengthier deliberations on true benefit and appropriateness for funding. These deliberations would impact review timelines and would make the drug more likely to receive a negative recommendation for funding.

In a study conducted by Gotfrit et al. to examine the determinants of cancer drug funding in Canada, the authors found an association between pCODR recommendations, cancer type, and drug class in determining the time to funding (TTF), which was defined as Health Canada approval to first provincial funding [16]. Tumour type was predictive of TTF (*p* < 0.001) at the provincial level with colorectal cancer (CRC) drugs being the slowest and neuroendocrine tumours (NETs) being the quickest. Our study found that drug class and tumour type do not impact final health technology recommendation at the national level, which may signify that the delay is potentially due to the variability in timelines from pCODR final recommendation to provincial funding. Prior studies have shown that it can take up to 5 to 11 months for a cancer drug that has been recommended for funding to be placed on provincial cancer formularies [17]. Despite a favourable pCODR recommendation, provincial funding delays could be due to factors such as differences in the provincial budget by tumour site or differential provincial priorities by tumour site. Provincial priorities are often driven by unmet needs and patient advocacy groups, which may indicate why some tumour sites with more unmet needs or larger and more vocal patient advocacy groups may have faster provincial funding of new cancer medicines. 

The balance between shortening approval times, allowing oncology patients to receive therapies earlier, and ensuring treatments are safe and clinically effective is a delicate one that all regulatory bodies are faced with [18]. In the United States, there is an accelerated approval process for certain medicines. Canada does not have an accelerated approval process for cancer medicines, and due to the nature of the healthcare system, cost considerations must also be factored heavily into funding decisions. Concerns about the efficacy of oncology therapies authorized by accelerated review and surrogate endpoints have been raised, as only 20% of drugs approved with a surrogate endpoint later showed improvement in overall survival [19].

This study relied on the accuracy of the information available on CADTH. Our study was limited by the small number of drugs that have been reviewed in the past, particularly when subdivided into the different tumour types and/or drug classes. Missing incremental cost-effectiveness ratio data also limited our ability to consider the economic impact of a new cancer medicine in its review time. This is an important variable that undoubtedly causes more deliberation and thus results in delays when the cost-effectiveness ratio is not favourable. Despite missing economic data, our analyses controlled for many of the known variables that could impact funding recommendations. 

## 5. Conclusions

In summary, our study demonstrates an association between longer drug review times and negative funding recommendations by pCODR. Although these recommendations and review times are not impacted by drug type or tumour type, we found that GI and lung cancer drugs tended to have longer review timelines. This study identifies drug review times as a factor in ultimate public funding recommendations. Further research will investigate factors that impact these timeline variations and streamline review processes to provide efficacious treatment to patients in a timely manner. 

## Figures and Tables

**Table 1:** Median time (in days) from Health Canada approval to final recommendation by tumour type. GU, genitourinary; GI, gastrointestinal.

Tumour Type	Median Time from Health Canada Approval to Final Recommendation	Mean Time from Health Canada Approval to Final Recommendation	*p*-Value (Kruskal–Wallis)
Lung cancer (N = 24)	198	299	0.0312
Breast cancer (N = 16)	111	214	
Other cancers (N = 89)	181	289	
GU (N = 17)	129	209	
GI (N = 18)	205	297	

**Table 2:** Median time (in days) from pCODR submission approval to final recommendation by tumour type. GU, genitourinary; GI, gastrointestinal.

Tumour Type	Median Length of Review in Days (from pCODR Submission to Final Recommendation)	Mean Length of Review in Days (from pCODR Submission to Final Recommendation)	*p*-Value (Kruskal–Wallis)
Lung cancer (N = 24)	223	236	0.1394
Breast cancer (N = 16)	212	221	
Other (N = 89)	202	207	
GU (N = 17)	203	204	
GI (N = 18)	204	214	

**Table 3:** Final pan-Canadian Oncology Drug Review (pCODR) recommendation by class of drug.

	Recommended/Conditionally Recommended for Funding	Not Recommended for Funding	Fisher’s Exact *p* Value
Cytotoxic chemotherapy	10	3	0.55
Targeted therapy	90	27	
Immunotherapy	24	4	
Other mechanism of action	6	0	

## Data Availability

The data presented in this study are available on request from the corresponding author.

## Supplementary Materials

The following supporting information can be downloaded at: https://www.mdpi.com/article/10.3390/curroncol30080558/s1, Table S1: Estimates of the mean length of review from pCODR submission to recommendation (LOR-SUB) for different cancer types compared to BC from the multiple linear regression model with p-value and 95% confidence intervals. pCODR, pan-Canadian Oncology Drug Review; GI, gastrointestinal; BC, breast cancer; GU, genitourinary; Table S2: Estimates of the mean length of review from Health Canada Notice of Compliance to pCODR recommendation (LOR-NOC) for different cancer types compared to BC from the multiple linear regression model with p-value and 95% confidence intervals. pCODR, pan-Canadian Oncology Drug Review; GI, gastrointestinal; BC, breast cancer; GU, genitourinary.
