# Supplementary material for: Impact of Oncology Drug Review Times on Public Funding Recommendations
Source: Curr Oncol. 2023 Aug 18;30(8):7706–12. doi: 10.3390/curroncol30080558 (PMC10453657; doi:10.3390/curroncol30080558)
Supplement: Supplementary file 1 [file curroncol-30-00558-s001.zip › curroncol-2428808-supplementary.pdf]

Supplementary Table 1: Estimates of the mean length of review from pCODR submission to recommendation (LOR-SUB) for different cancer types compared to BC from the multiple linear regression model with p-value and 95% confidence intervals. pCODR, pan-Canadian Oncology Drug Review. GI, gastrointestinal; BC, breast cancer; GU, genitourinary.

**Cancer type**	**Estimate**	**p-value (95% CI)**
GI vs BC	-10.3	0.52 (-41.8, 21.2)
GU vs BC	-15.7	0.33 (-47.8, 16.4)
Lung vs BC	14.4	0.34 (-15.6, 44.4)
Others vs BC	-14.7	0.24 (-39.2, 9.8)

Supplementary Table 2: Estimates of the mean length of review from Health Canada Notice of Compliance to pCODR recommendation (LOR-NOC) for different cancer types compared BC from the multiple linear regression model with p-value and 95% confidence intervals. pCODR, pan-Canadian Oncology Drug Review. GI, gastrointestinal; BC, breast cancer; GU, genitourinary.

**Cancer type**	**Estimate**	**p-value (95% CI)**
GI vs BC	1.55	0.11 (0.91, 2.64)
GU vs BC	1.24	0.42 (0.73, 2.11)
Lung vs BC	1.74	0.03 (1.06, 2.87)
Others vs BC	1.57	0.03 (1.04, 2.37)
Recommended (Yes vs No)	0.55	<0.001 (0.41, 0.74)
